# MythPose: Enhanced Detection of Complex Poses in Thangka Figures

**DOI:** 10.3390/s25164983

**Published:** 2025-08-12

**Authors:** Yukai Xian, Te Shen, Yurui Lee, Ping Lan, Qijun Zhao, Liang Yan

**Affiliations:** 1 School of Information Science and Technology, Tibet University, Lhasa 850000, China; yukai@stu.utibet.edu.cn (Y.X.);; 2 School of Humanities, Tibet University, Lhasa 850000, China; 3 College of Computer Science, Sichuan University, Chengdu 610065, China

**Keywords:** deep learning, human pose estimation, Thangka, YOLO, MythPose

## Abstract

Thangka is a unique form of painting in Tibet, which holds rich cultural significance and artistic value. In Thangkas, in addition to the standard human form, there are also figures with multiple limbs. Existing human pose estimation methods are not well suited for keypoint detection of figures in Thangka paintings. This paper builds upon YOLOv11-Pose and introduces the Mamba structure to enhance the model’s ability to capture global features. A feature fusion module is employed to integrate both shallow and deep features, and a KAL loss function is proposed to alleviate the interference between keypoints of different body parts. In this study, a dataset of 6208 Thangka images is collected and annotated for Thangka keypoint detection, and data augmentation techniques are used to enhance the generalization of the dataset. Experimental results show that MythPose achieves 89.13% mAP@0.5, 92.51% PCK, and 87.22% OKS in human pose estimation tasks on Thangka images, outperforming the baseline model. This research not only provides a reference for the digital preservation of Thangka art but also offers insights for pose estimation tasks in other similar artworks.

## 1. Introduction

Thangka is a unique artistic form originating from Tibet, characterized by varied postures, diverse styles, and vibrant colors, while also adhering to strict proportional requirements in its depiction. Most Thangkas are portrait-style, where the central figure, often the principal deity, is the largest object in the image, with other figures or decorative elements arranged around it. Thangka paintings are rich in cultural significance, representing a product of interaction and integration between the Tibetan people and other ethnic groups, and they hold high historical and cultural value. The use of digital technology to preserve and develop artworks has become a popular topic. Many computer researchers have also attempted to apply technologies such as image classification and image recognition in computer vision to study Thangka.

Some researchers have made significant contributions in dataset construction and evaluation, providing valuable references for the work of future scholars. Ma et al. [1] constructed the CYTKv1 dataset, which contains over 1700 Thangka images, by collecting Thangka images and manually annotating them. To address the challenge of evaluating the quality of Thangka image restoration, Hu et al. [2] proposed a no-reference method based on symmetry and structural characteristics, using Generative Adversarial Networks (GANs). This approach also offers valuable insights for building larger-scale Thangka datasets. In addition, Xian et al. [3] introduced a region-aware style transfer framework for Thangka images, combining segmentation with adaptive style fusion to preserve both regional structure and artistic characteristics, which also provides methodological inspiration for downstream tasks such as human pose estimation.

Subsequent studies have integrated technologies such as segmentation, matching, and retrieval from computer vision, and have improved certain methods to better adapt to the visual tasks in Thangka images based on their unique characteristics. In 2021, Wang et al. [4] conducted research on semantic segmentation in Thangka paintings, enhancing feature extraction and segmentation accuracy by using convolutional kernels of various sizes to expand the receptive field. In the context of cultural heritage image analysis, Shen et al. [5] developed a lightweight semantic feature-based algorithm for ancient mural element object detection, demonstrating the effectiveness of deep learning approaches for complex heritage artworks. Xian et al. [6] proposed a multi-feature fusion approach for object detection in Thangka images, incorporating Gabor, wavelet, and color features to address the challenges posed by the intricate patterns and diverse styles found in Thangka artworks. In the field of Thangka image retrieval, Li et al. [7] explored matching Thangka sketches with finished works to improve retrieval accuracy and the model’s understanding of Thangka content. Building on this, Xian et al. [8] introduced a multi-attribute feature learning framework, further advancing Thangka school image retrieval by enabling more precise categorization and retrieval of different Thangka styles.

For the recognition and matching of figures in Thangka paintings, these methods can assist researchers in better analyzing the representation of characters across different Thangkas. Yang et al. [9] proposed an MGFL model, which extracts features at different granularities and performs fusion to improve the accuracy of figure recognition in Thangka images. To enhance the accuracy of extracting facial features from figures in Thangkas, Yang et al. [10] used the Dlib library to recognize facial keypoints in Thangka characters. Using digital methods to restore damaged Thangkas can help reproduce the original appearance of the artwork, but finding the best approach to digital restoration remains a highly challenging task. Hsieh et al. [11] proposed a two-stage framework to improve the restoration of damaged Thangkas. This framework is based on diffusion models and GANs, using text and edge information to guide the model in restoring the Thangka. This method not only preserves the visual consistency of the restored areas but also enhances the level of detail in the restoration.

These studies have made significant contributions in the segmentation, recognition, and retrieval of Thangka images, greatly promoting the digital preservation and development of Thangka art, while also providing feasible reference methods for future researchers. Human pose estimation algorithms can help recognize body postures by detecting keypoints, and the figures in Thangkas carry rich cultural connotations. Using human pose estimation algorithms to study the postures of figures in Thangka paintings holds important significance. However, there is still a lack of research on applying human pose estimation technology to the recognition and detection of figures in Thangka images. There are several challenges in estimating human poses in Thangka images, making research progress in this area difficult. In addition to normal human forms, many figures in Thangka paintings have a number of limbs beyond the typical range. Generally speaking, the number of keypoints should be fixed, but the variable number of limbs makes it difficult to pre-define the number of keypoints. Furthermore, many figures in Thangkas wear loose robes, which adds complexity to keypoint annotation and detection by the model.

To address the unique challenges of pose estimation in Thangka art, this study constructed a dedicated human pose estimation dataset for Thangka images, with expert collaboration ensuring the reliability and quality of annotations. Building upon YOLOv11-Pose, we introduced the Mamba structure to enhance global feature extraction and incorporated a feature fusion module to effectively combine shallow and deep features. Furthermore, we propose a body affinity field to alleviate the difficulties of keypoint association across multiple limbs. We name our method MythPose, as it is specifically designed to handle the mythological multi-limb characteristics frequently observed in Thangka figures, where deities or symbolic beings often have complex postures and additional arms, posing unique challenges for conventional pose estimation models. Our research not only fills a critical gap in Thangka human pose estimation, providing a solid foundation for future visual analysis in this domain, but also offers methodological insights that are potentially transferable to related fields such as religious and mural art, which share similar visual and structural characteristics. Although the current lack of publicly available annotated datasets for other artistic traditions limits direct cross-domain validation, we hope this study will inspire further research on pose estimation and structural analysis in diverse artistic and cultural heritage contexts.

## 2. Related Work

### 2.1. Pose Estimation

In recent years, deep learning has made remarkable progress in image processing and visual recognition. Pose estimation in 2D images, especially for human figures, is a highly valuable research task. Traditional 2D pose estimation can be divided into single-person and multi-person pose estimation tasks.

For single-person pose estimation, current methods fall into three categories: regression-based methods, heatmap-based methods, and hybrid approaches. Regression-based methods predict keypoint coordinates of the human body and are known for their simplicity, directness, and efficiency. In 2014, Toshev and Szegedy [12] first introduced deep learning to pose estimation, significantly improving prediction accuracy. In 2019, Luvizon et al. [13] employed a softmax operation to convert heatmaps into coordinate predictions, achieving end-to-end training. In 2021, Mao et al. [14] and Li et al. [15] enhanced feature extraction and accuracy by incorporating Transformers and using cascaded Transformers, respectively. In 2022, Mao et al. [16] treated pose estimation as a sequence prediction task with the Poseur model, improving accuracy and robustness.

Heatmap-based methods estimate keypoint probabilities for each pixel. In 2017, Sun et al. [17] introduced a two-stage normalization scheme that simplified model learning and improved accuracy. In 2017, Marras et al. [18] enhanced joint position estimation by introducing geometric constraints with a spatial model network. In 2022, Ke et al. [19] employed a structure-aware network to accurately capture human body structures, and in 2018, Tang et al. [20] utilized a deep learning compositional model to improve pose estimation efficiency. The data grouping approach by Tang and Wu [21] and the SimCC method by Li et al. [22] each improved performance through grouped learning and task transformation.

In regression combined with heatmap-based methods, Li et al. [23] proposed a human pose regression method in 2021 using residual log-likelihood estimation, enhancing the accuracy and reliability of pose estimation tasks. In 2023, Ye et al. [24] employed a token-distillation encoder and simulated heatmap to transfer heatmap-based knowledge from a high-performance teacher model to a regression-based student model, integrating the strengths of both models to improve pose estimation efficiency and accuracy.

In multi-person pose estimation tasks, existing methods can be divided into three types: top-down, bottom-up, and single-stage approaches. Top-down methods typically execute in two stages: first detecting each person in the image, followed by individual pose estimation for each detected person. In 2019, Sun et al. [25] used a High-Resolution Network (HRNet) to enhance the accuracy of pose estimation. Su et al. [26] introduced the Channel Shuffle Module and Spatial-Channel Attention Residual Bottleneck to improve estimation in occluded scenes. In 2021, Yang et al. [27] developed TransPose, and Li et al. [28] introduced TokenPose, both leveraging CNNs and tokenized representations to model global relationships and estimate occluded keypoint locations. The same year, Wang et al. [29] proposed HRFormer, a Transformer module that fuses multi-resolution features to improve the accuracy of dense pose estimation. In 2022, Xu et al. [30] demonstrated the capabilities of the Vision Transformer architecture with ViTPose.

Bottom-up methods first detect all human keypoints and then associate them to form complete poses. In 2017, Cao et al. [31] introduced OpenPose, which used Part Affinity Fields to encode limb position and orientation. In 2019, Kreiss et al. [32] proposed using Part Intensity Fields, while Li et al. [33] created the CrowdPose benchmark. In 2020, Cheng et al. [34] addressed scale variation issues using HigherHRNet with deconvolution. In 2021, Geng et al. [35] developed Disentangled Keypoint Regression to learn representations that focus on keypoint regions. In 2023, Li et al. [36] introduced PolarPose, simplifying 2D regression in polar coordinates.

Single-stage methods integrate human detection and pose estimation into a single network. In 2019, Tian et al. [37] introduced DirectPose, which eliminated grouping post-processing. In 2021, Shi et al. [38] proposed InsPose, which adaptively adjusted parameters to enhance performance flexibility. In 2022, Shi et al. [39] presented PETR, viewing pose estimation as a set prediction task, creating the first fully end-to-end framework. In 2023, Miao et al. [40] developed the Single-Stage Multi-Person Pose Regression network to reduce false-positive poses. Yang et al. [41] proposed ED-Pose, which forms an end-to-end framework through a decoder cascade, while Liu et al. [42] used a simple Transformer decoder in Group Pose to pursue efficiency.

Traditional pose estimation tasks are typically trained and fine-tuned on specific category datasets, a process that is often costly. In 2022, Xu et al. [43] introduced a new task called Category-Agnostic Pose Estimation, aiming to create a model capable of detecting the pose of any object category using keypoint definitions from only a small number of samples. They framed pose estimation as a keypoint matching problem and designed the POMNet network architecture. Additionally, they introduced a 2D pose dataset, Multi-category Pose (MP-100), containing over 20,000 instances across 100 object categories, specifically designed to support the development of CAPE algorithms. In the same year, Min Shi et al. [44] addressed the accuracy limitations of traditional single-stage CAPE methods by introducing a new two-stage framework. This framework first generates similarity-aware position proposals by matching keypoints, and then it refines the initial proposals in the second stage by invoking relevant features. Their custom-designed Transformer encoder model improved representation and similarity modeling in the first matching stage. In the second stage, similarity-aware proposals are refined in the decoder as queries, improved through cross-attention. This approach significantly surpassed previous methods in both accuracy and efficiency on the MP-100 dataset. In 2023, Wang et al. [45] introduced a novel Graph Transformer Decoder, which utilizes the inherent geometric relationships between keypoints to significantly enhance keypoint localization accuracy. This innovation marked a major shift from the traditional CAPE approach, which treats keypoints as isolated entities. Moreover, the end-to-end training capability of this method demonstrated better scalability and efficiency compared to previous CAPE methods.

### 2.2. YOLOv11-Pose

The YOLOv11-Pose framework is an efficient single-stage detection model designed for human pose estimation tasks. It integrates both keypoint localization and object detection functions while adopting a lightweight design and efficient inference mechanism, ensuring high accuracy along with fast inference speeds.

The overall architecture of YOLOv11-Pose follows the same structure as YOLOv11, consisting of a backbone network, a neck, and detection heads. The backbone includes clear convolutional layers and C3k2 modules, which enable YOLOv11-Pose to quickly extract fine-grained features from images and pass them to subsequent modules for further processing, providing richer feature representations for the pose estimation task. The neck employs a PAN structure that uses a series of upsampling and downsampling operations to obtain features at different granularities. These features are then fused through concatenation, enhancing the accuracy of keypoint detection at various scales. YOLOv11-Pose uses different types of detection heads to perform tasks such as bounding box classification, bounding box regression, and keypoint detection. These heads can independently complete their respective tasks and also cooperate to improve the accuracy of other tasks.

During the training phase, YOLOv11-Pose employs several data augmentation methods, including color jitter, adaptive image scaling, and Mosaic, to increase the diversity of the dataset and improve the model’s generalization capability, enabling it to perform well in various complex scenarios. Additionally, YOLOv11-Pose jointly utilizes multiple loss functions to optimize the learning process. These loss functions include traditional regression losses for detection tasks and specialized losses designed specifically for keypoint detection, optimizing the overall performance of pose estimation.

### 2.3. Mamba

Mamba [46] is an efficient linear time-series framework that combines a selective state-space model with a sequence model. Compared to the Transformer structure, Mamba offers higher computational efficiency and lower overhead in long sequence modeling tasks.

The selective state-space model in Mamba is a key component of the Mamba structure. It dynamically selects, retains, or ignores certain types of input information. Traditional selective state-space models (SSMs) struggle with handling data from complex modalities, but Mamba overcomes this limitation by introducing a selection mechanism to filter out key information. The selection mechanism in Mamba works by dynamically adjusting the state transition matrix A, input matrix B, output matrix C, and timestep Δ in the SSM. This approach allows Mamba to discard irrelevant information, retain important details, and propagate them effectively.

Let h(t) represent the hidden state, x(t) the input, and y(t) the output. The basic formula for the SSM is as follows:

(1) $$\begin{array}{cc} h(t)=Ah(t)+Bx(t), & y(t)=Ch(t) \end{array}$$

The Mamba structure implements content-based selective propagation, a mechanism that enables Mamba to handle different modalities of information—such as text, audio, and even images—more flexibly. However, the introduction of the selective mechanism also presents computational challenges. To address this, researchers have designed a hardware-aware parallel recursive algorithm to improve the efficiency of the Mamba structure. With the combined effect of the parallel recursive algorithm and the selective mechanism, Mamba can achieve high-precision sequence modeling without sacrificing efficiency. Its inference speed is three times faster than traditional SSM methods, and its generation speed is five times faster than Transformer models with the same number of parameters.

The innovative design of the Mamba structure allows it to excel in long sequence modeling tasks, such as text processing and audio modeling. In image processing tasks, Mamba further enhances its computational efficiency by incorporating a unique selective mechanism. This mechanism enables the model to selectively focus on important regions based on the input features, improving feature extraction accuracy while reducing computational complexity. Not only does this selective mechanism significantly reduce the computational resource consumption during training and inference, but it also maintains high performance when processing high-resolution images.

## 3. Methods

Traditional human pose estimation algorithms perform well in single- or multi-person keypoint detection tasks, but they cannot be directly applied to the scenario of multiple limbs in single figures, as seen in Thangka images. This study addresses the issue of multiple limbs in the central deity figure in Thangkas by improving the YOLOv11-Pose algorithm, making it better suited for human pose estimation tasks in Thangka images.

**Figure 1:**
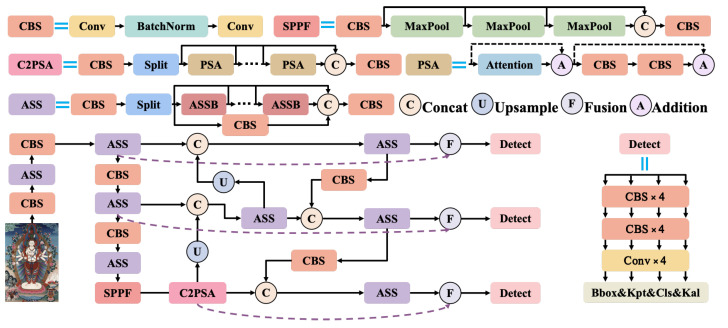
The MythPose model improves upon YOLOv11 by replacing the bottleneck blocks within the C3k2 modules with the proposed ASSB modules featuring a Mamba structure. In addition, a feature fusion module is added before the detection head, and the KAL loss is integrated into the detection head to further enhance performance.

The traditional YOLOv11-Pose algorithm boasts high efficiency and accuracy. However, the C module used for feature extraction has significant limitations in extracting and selecting global information, with a stronger focus on local details. To enable the model to capture more global features, a common approach is to introduce Transformer structures, which leverage self-attention and multi-head attention mechanisms. Transformers bring high computational overhead and reduce the efficiency of YOLOv11-Pose. Our model MythPose (Figure 1) uses a State Space Model (SSM) with a selective mechanism to capture long-range information more efficiently. We designed an Advanced Scanning and Synthesis (ASS) module based on the Mamba structure to replace the original C3k2 modules. This improves global feature extraction while keeping the computation low. We also added a feature fusion stage before the detection head to combine multi-scale features. In addition, we used KAL loss to improve the accuracy of keypoint localization.

Furthermore, to integrate shallow features containing more local detail with deeper features that carry more global semantic information, this study introduces a fusion module before the detection head of YOLOv11-Pose. This module, combining DPA and SAA mechanisms, effectively captures global and spatial contextual information, enhances the complementarity of features, and provides the detection head with richer feature information.

To better address the issue of multiple limbs overlapping and occluding each other in the central deity of Thangka paintings, we designed a keypoint association loss function to optimize the model’s ability to distinguish between limbs, improving the classification accuracy of limb keypoints. It also enables the model to establish both local keypoint connections for individual limbs and global connections for the entire figure.

These methods, while maintaining the high efficiency of YOLOv11-Pose, offer a more flexible approach to dealing with the challenges in Thangka images, where the number of limbs in the central deity is unclear and limbs may obscure each other.

### 3.1. Advanced Scanning and Synthesis Block (ASSB)

**Figure 2:**
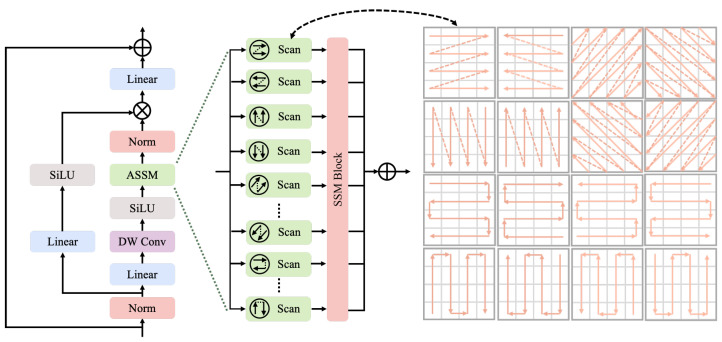
The ASSB builds upon the Mamba structure by incorporating an enhanced SSM block. The modifications involve adding multiple scanning strategies to improve spatial feature extraction and adaptability. Key components include SiLU activation, depthwise convolution (DW Conv), and normalization layers, working together to refine and optimize feature representations. The right section illustrates the diverse scanning patterns integrated into the SSM block, demonstrating the module’s ability to capture complex spatial dependencies.

In enhancing YOLOv11, the original C3k2 module, known for its efficient feature fusion and reuse capabilities, is particularly suitable for lightweight networks. However, it exhibits limitations in complex scenes, especially in capturing long-range dependencies and multi-scale feature representation. To address this, we propose replacing the C3k2 module with the Mamba structure (Figure 2). By incorporating the SSM, the Mamba structure excels at modeling long-range dependencies and capturing global information, making it particularly effective for tasks involving long sequence processing and enhancing global perception capabilities.

Despite its advantages, the traditional Mamba structure’s simple scanning mechanism, which is limited to a single mode, restricts its flexibility and expressive power in complex feature extraction tasks. To overcome this limitation, we improve the SSM module by introducing multiple scanning directions. This enhancement increases the flexibility of the scanning function, enabling it to capture features from various angles and orientations, which is crucial for representing intricate patterns in Thangka images.

The scanning process begins with multi-layer linear transformations for feature mapping. This step projects the input into a higher-dimensional feature space, facilitating the separation of meaningful spatial cues. Assuming the input feature is $F_{\mathrm{in}}$, it undergoes the first linear transformation as follows:

(2) $$F_{\mathrm{in}}^{\left(1\right)}=\mathrm{Linear}\left(F_{\mathrm{in}}\right)$$

Here, $F_{\mathrm{in}}^{\left(1\right)}$ denotes the initial transformed feature. The feature is then subjected to normalization and nonlinear activation, which stabilizes the feature distribution and introduces nonlinearity, enabling the model to better capture complex spatial relationships. The index *i* indicates the intermediate step in the transformation pipeline, where each stage can be interpreted as a progressive refinement of the feature representation:

(3) $$F_{\mathrm{norm}}^{\left(i\right)}=\mathrm{Norm}\left(F_{\mathrm{in}}^{\left(i\right)}\right)$$

(4) $$F_{\mathrm{act}}^{\left(i\right)}=\mathrm{SiLU}\left(F_{\mathrm{norm}}^{\left(i\right)}\right)$$

Following this, a depthwise separable convolution (DW Conv) further refines the feature representation, allowing the module to emphasize local structure and subtle details that are important for keypoint localization:

(5) $$F_{\mathrm{dw}}=\mathrm{DWConv}\left(F_{\mathrm{act}}^{\left(i\right)}\right)$$

The feature then undergoes an additional layer of linear transformation and normalization, which aggregates information from previous steps and prepares the feature for subsequent directional scanning. The index *o* represents the output stage:

(6) $$F_{\mathrm{out}}^{\left(o\right)}=\mathrm{Linear}\left(F_{\mathrm{dw}}\right)$$

(7) $$F_{\mathrm{norm}}^{\left(o\right)}=\mathrm{Norm}\left(F_{\mathrm{out}}^{\left(o\right)}\right)$$

Finally, these processed features are input into the SSM module, which performs scanning operations in multiple directions. By applying horizontal, vertical, diagonal, and reverse-diagonal scans, the network can effectively aggregate spatial information from various orientations, enhancing its ability to model complex pose structures and symmetries in Thangka images:

(8) $$F_{\mathrm{scan}}=\mathrm{SSM}\left(\sum_{d\in D}\mathrm{Scan}d\left(F{\mathrm{out}}^{\left(o\right)}\right)\right)$$

where *D* represents the set of all scanning directions.

By incorporating multiple directional scans, our improvement allows the SSM module to more comprehensively address features of varying dimensions and orientations, significantly enhancing feature representation in complex scenes. This contributes to improved detection performance in YOLOv11, especially for intricate and occluded features found in Thangka art.

### 3.2. Fusion Module

**Figure 3:**
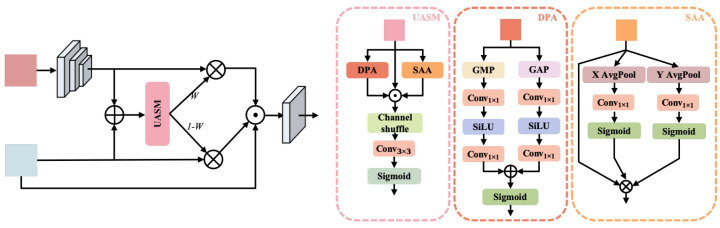
The **left** diagram presents the overall fusion structure, which integrates features using adaptive weighting and multiple aggregation paths. One key component within this structure is the Unified Adaptive Spatial Module (UASM), depicted in the detailed panels on the **right**. The UASM includes Dual Pooling Attention (DPA), which uses global max and average pooling with SiLU activation for effective attention generation, and Spatial Axis Attention (SAA), which captures spatial dependencies through directional average pooling. These modules work together, incorporating channel shuffling and a 3 × 3 convolution, to enhance the fusion process adaptively.

The overall structure of the proposed module, as shown in Figure 3, starts with the fusion of low-dimensional features $F_{L}$ (closer to the input, capturing more local detail) and high-dimensional features $F_{H}$ (from deeper layers, encoding more abstract global information). The low-dimensional feature map $F_{L}$ is processed through a convolutional layer to align it with the dimensionality of $F_{H}$:

(9) $$F_{\mathrm{aligned}}={\mathrm{Conv}}_{3\times 3}\left(F_{L}\right)$$

The aligned low-dimensional features are then combined with the high-dimensional features through element-wise addition, resulting in a fused feature representation:

(10) $$F_{\mathrm{fusion}}=F_{\mathrm{aligned}}+F_{H}$$

The fused feature $F_{\mathrm{fusion}}$ is then fed into the UASM, which employs both DPA and SAA mechanisms to extract comprehensive attention features. The DPA module uses global max pooling (GMP) and global average pooling (GAP) to extract global contextual information from $F_{\mathrm{fusion}}$, generating feature vectors $F_{\mathrm{GMP}}$ and $F_{\mathrm{GAP}}$:

(11) $$F_{\mathrm{GMP}}=\mathrm{GMP}\left(F_{\mathrm{fusion}}\right);\;F_{\mathrm{GAP}}=\mathrm{GAP}\left(F_{\mathrm{fusion}}\right)$$

These feature vectors are passed through 1 × 1 convolutions followed by the SiLU activation function and then processed with a Sigmoid function to produce channel attention weights:

(12) $$W_{\mathrm{GMP}}=\mathrm{Sigmoid}\left({\mathrm{Conv}}_{1\times 1}\left(\mathrm{SiLU}\left(F_{\mathrm{GMP}}\right)\right)\right);\;W_{\mathrm{GAP}}=\mathrm{Sigmoid}\left({\mathrm{Conv}}_{1\times 1}\left(\mathrm{SiLU}\left(F_{\mathrm{GAP}}\right)\right)\right)$$

The final channel attention map $W_{\mathrm{DPA}}$ is obtained by combining these weights:

(13) $$W_{\mathrm{DPA}}=W_{\mathrm{GMP}}+W_{\mathrm{GAP}}$$

Simultaneously, the SAA module captures spatial distribution by performing average pooling along the X and Y axes to generate spatial feature maps $F_{X}$ and $F_{Y}$:

(14) $$F_{X}=\mathrm{XAvgPool}\left(F_{\mathrm{fusion}}\right);\;F_{Y}=\mathrm{YAvgPool}\left(F_{\mathrm{fusion}}\right)$$

These feature maps are processed through 1 × 1 convolutions and activated using Sigmoid functions to produce spatial attention weights:

(15) $$W_{X}=\mathrm{Sigmoid}\left({\mathrm{Conv}}_{1\times 1}\left(F_{X}\right)\right);\;W_{Y}=\mathrm{Sigmoid}\left({\mathrm{Conv}}_{1\times 1}\left(F_{Y}\right)\right)$$

The final spatial attention map $W_{\mathrm{SAA}}$ is obtained by combining these weights:

(16) $$W_{\mathrm{SAA}}=W_{X}+W_{Y}$$

The attention features $W_{\mathrm{DPA}}$ and $W_{\mathrm{SAA}}$ are concatenated along the channel dimension together with $F_{\mathrm{fusion}}$ and then subjected to a channel shuffle operation to promote information interaction among different channels:

(17) $$F_{\mathrm{concat}}=\mathrm{Concat}\left(W_{\mathrm{DPA}},W_{\mathrm{SAA}},F_{\mathrm{fusion}}\right)$$

(18) $$F_{\mathrm{shuffle}}=\mathrm{ChannelShuffle}\left(F_{\mathrm{concat}}\right)$$

The shuffled features are processed through a 3 × 3 convolution and a Sigmoid activation function to generate new attention weights:

(19) $$W=\mathrm{Sigmoid}({\mathrm{Conv}}_{3\times 3}\left(F_{\mathrm{shuffle}}\right))$$

These attention weights *W* are then used to adjust the low-dimensional feature map $F_{L}$ and the high-dimensional feature map $F_{H}$ through element-wise multiplication, enhancing feature representation by emphasizing the most relevant regions:

(20) $$F_{\mathrm{weighted}1}=W\cdot F_{L},\;F_{\mathrm{weighted}2}=\left(1-W\right)\cdot F_{H}$$

Finally, $F_{\mathrm{weighted}1}$ and $F_{\mathrm{weighted}2}$ are concatenated with the original high-dimensional feature map $F_{H}$ to produce the output feature:

(21) $$F_{\mathrm{output}}={\mathrm{Conv}}_{1\times 1}\left(\mathrm{Concat}\left(F_{H},F_{\mathrm{weighted}1},F_{\mathrm{weighted}2}\right)\right)$$

By incorporating both DPA and SAA mechanisms within the UASM, the module effectively captures global contextual and local spatial information. The design ensures that essential features are emphasized while maintaining a robust representation that adapts well to complex scenes and diverse structures. This combination significantly enhances the model’s performance in keypoint detection and other tasks requiring fine-grained feature understanding.

### 3.3. Loss Function

**Figure 4:**
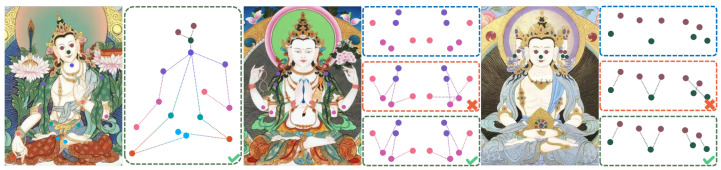
Keypoint connection strategies for different figures. The **left** group shows the correct keypoint connections for a standard human body. The **middle** group illustrates connection strategies for multi-armed figures, with the blue box showing unconnected keypoints, the red box indicating incorrect connections, and the green box displaying the correct connections. The **right** group presents connection strategies for multi-headed figures, using the same color coding to represent unconnected, incorrect, and correct connections.

In Thangka images, where the number of limbs in the central deity is unclear and limbs may overlap or obscure each other, this study designed a Keypoint Association Loss (KAL) to improve the model’s ability to distinguish between keypoints and correctly group them. This is particularly important in Thangka images, where, as shown in Figure 4, unclear groupings often lead to ambiguous or incorrect keypoint connections. The KAL loss function helps the model more accurately judge the dependencies between keypoints, resulting in more precise keypoint associations and improved overall pose estimation performance.

Our approach introduces embedding vectors and association losses for keypoints. In the embedding space, keypoint features are learned so that distances between keypoints from the same figure are minimized, while distances between keypoints from different figures are increased. For a detected keypoint *i*, we obtain its embedding $e_{i}\in R^{D}$ (e.g., a feature vector from a global feature extraction network), where *D* is the dimensionality of the embedding. Based on annotated labels, the association label $y_{ij}$ between keypoints *i* and *j* is defined as follows:

(22) $$y_{ij}=\left\{\begin{array}{cc} 1, & \mathrm{if}\;\mathrm{keypoints}\;i\;\mathrm{and}\;j\;\mathrm{should}\;\mathrm{be}\;\mathrm{associated},\\ 0, & \mathrm{otherwise}. \end{array}\right.$$

To measure the distance relationship between embedded keypoints, we adopt a contrastive loss function defined as follows:

(23) $$L_{KAL}=\frac{1}{N_{\mathrm{pos}}}\sum_{(i,j)\in P}∥e_{i}-e_{j}{∥}_{2}^{2}+\frac{1}{N_{\mathrm{neg}}}\sum_{(i,j)\in N}\max(0,m-∥e_{i}-e_{j}{{∥}_{2})}^{2},$$

where *P* represents the set of positive pairs (i.e., $y_{ij}=1$), *N* represents the set of negative pairs (i.e., $y_{ij}=0$), and $N_{\mathrm{pos}}$ and $N_{\mathrm{neg}}$ denote the numbers of positive and negative samples, respectively. The parameter *m* is a pre-defined margin to increase the separation distance between non-associated keypoints, and ${∥\cdot ∥}_{2}$ represents the Euclidean distance. This loss function aims to minimize the distances between associated keypoints in the embedding space, ensuring closer proximity, while maximizing the distances between non-associated keypoints to avoid erroneous associations.

The overall loss function for YOLOv11-Pose with the Keypoint Association Loss is defined as follows:

(24) $$L=\lambda_{\mathrm{bbox}}L_{\mathrm{bbox}}+\lambda_{\mathrm{cls}}L_{\mathrm{cls}}+\lambda_{\mathrm{kpt}}L_{\mathrm{kpt}}+\lambda_{KAL}L_{KAL},$$

where $L_{\mathrm{bbox}}$ is the bounding box loss, $L_{\mathrm{cls}}$ is the classification loss, $L_{\mathrm{kpt}}$ is the keypoint location loss, and $L_{KAL}$ is the keypoint association loss. The parameters $\lambda_{\mathrm{bbox}},\lambda_{\mathrm{cls}},\lambda_{\mathrm{kpt}},\lambda_{KAL}$ are weighting coefficients to balance the different components of the loss.

## 4. Experiments

### 4.1. Dataset

Due to differences in materials and periods, the preservation conditions of ancient Thangka paintings vary, resulting in a limited number of surviving works, many of which exhibit varying degrees of damage. To ensure both the quantity and quality of our dataset, we collected images from online sources, including digital Thangka websites and several open-source repositories. Additionally, authoritative books such as “Collection of Tibetan Art: Chamdo Volume” [47] and “Chinese Thangka” [48] were consulted to supplement background and contextual information.

The experimental data consists of a total of 6208 Thangka portrait images collected from these sources. The images were categorized based on the number of limbs of the figures into three types: normal, multi-headed, and multi-armed. In this study, we used the Labelme tool to annotate limb regions and keypoints within each region. The annotations were then converted to the YOLO dataset format. The keypoint categories include eyes, nose, neck, shoulders, elbows, wrists, hips, knees, and ankles.

The size of the dataset is a critical factor in model training, as larger datasets generally improve model performance. Given the unique characteristics of image data, effective data augmentation strategies must account for the specific distribution patterns within the dataset, combined with insights from practical experience, to create tailored augmentation methods. Such strategies not only expand the dataset but also enhance the diversity of data features. In this study, we explored and implement various data augmentation techniques specifically designed to leverage the unique attributes of Thangka images, aiming to improve the model’s generalization capability in Thangka figure pose estimation tasks.

To address common challenges in general image datasets, we initially applied traditional data augmentation techniques, including random grayscale, color jitter, random cropping, Mosaic [49], CutMix [50], random flipping, Gaussian blur, and random rotation. These techniques enable the model to better adapt to the complex and varied nature of Thangka images across different scenes, enhancing its robustness in diverse environments.

To further simulate the actual deterioration observed in ancient Thangka images, we specifically trained a CycleGAN [51] generator to produce Thangka images with random damage effects corresponding to the original images. The images generated by the CycleGAN generator can effectively simulate common issues found in real Thangka images, such as damage and fading, which enhances the model’s robustness in handling Thangka images with varying levels of preservation. This also improves the model’s adaptability in complex scenes. Additionally, in Thangka images, the central figure is typically depicted wearing a robe and seated or lying down, and the robe can obscure the limbs, such as the hands and legs. To address this, this paper proposes a data augmentation method. First, we establish a library of common robe texture images. Then, for each hand and leg region of the central figure in the annotated original dataset, we form an area by connecting the keypoints of the hands and legs. Next, we extend the left and right boundaries of this area horizontally by a distance *r*, creating a new region. Finally, a robe pattern is randomly selected and overlaid within this new region, simulating the situation in real Thangka images where the central figure is dressed in a robe.

### 4.2. Experimental Setup

The experiment used the PyTorch (version 2.2.0) deep learning framework, with YOLOv11-Pose chosen as the baseline model. The parameters for the backbone network were initialized using pre-trained weights from CSPDarknet on the COCO dataset, while the newly added modules were initialized with the Xavier [52] method. Training was conducted on a server equipped with an NVIDIA 3080Ti GPU for 300 epochs with a batch size of 32, using the Adam optimizer. All input images were resized to 640 × 640 and normalized during preprocessing to accelerate training and enhance generalization. For the overall loss function, the weighting coefficients were set to $\lambda_{\mathrm{bbox}}=2.0$, $\lambda_{\mathrm{cls}}=1.0$, $\lambda_{\mathrm{kpt}}=1.0$, and $\lambda_{\mathrm{KAL}}=1.5$, as determined by grid search on the validation set to balance detection precision and keypoint association accuracy. The higher weight for $\lambda_{\mathrm{KAL}}$ underscores the importance of accurate keypoint grouping, which is critical for handling the complex multi-limb and occluded scenarios typical of Thangka images. In our robe occlusion augmentation strategy, the expansion distance *r* for the simulated robe area is randomly selected within [0.05W,0.1W], where *W* is the image width. This percentage-based range is empirically determined from annotated Thangka images, ensuring that the simulated occlusions realistically reflect actual limb coverage by robes while providing sufficient variability to improve model robustness during training.

### 4.3. Processing Analysis

**Figure 5:**
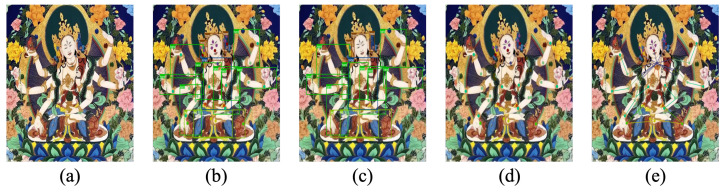
The figure illustrates the model’s comprehensive approach to processing a Thangka image with mythological features. (**a**) The original input image, rich in artistic and structural complexity. (**b**) The combined detection results, where the model simultaneously generates bounding boxes for key body regions—head, arms, torso, and legs—and identifies keypoints within each area. (**c**) This sub-figure focuses solely on the target detection boxes, which help localize and constrain the keypoint search regions. (**d**) The detected keypoints without the bounding boxes, pinpointing crucial anatomical landmarks such as elbows, eyes, and knees. (**e**) The final skeletal structure, where the keypoints are connected in the correct sequence, forming a cohesive and anatomically accurate representation of the deity’s pose.

This section illustrates the step-by-step process of how the model handles portrait-style Thangka images with mythological features, along with the processing results at each stage. Figure 5 visualizes the experimental outcomes, from the original input to the final pose estimation.

The model receives the input Thangka image and performs an initial analysis, simultaneously generating target detection boxes for limb regions and predicting keypoints within each area. The detection boxes encompass four categories: head, arms, torso, and legs, constraining the keypoints to their respective regions. Within each detection box, the model employs a keypoint detection module to identify the precise locations of keypoints, labeling them as elbows, eyes, knees, and so on. Finally, the model connects the keypoints of each limb region in a predefined sequence, linking them to the neck keypoint to form a complete skeletal structure. The experimental results show that the proposed method effectively addresses the challenge of indeterminate limb counts in Thangka figures, providing valuable insights for pose estimation research on other artworks with mythological features.

### 4.4. Comparative Experiment

This study conducted a series of experiments to validate the effectiveness of MythPose for human pose estimation in Thangka images. We evaluated our model on two representative categories: conventional human figures and the multi-limb “Four-Armed Avalokiteshvara” class. Seven classical keypoint detection models were selected as baseline models for comparative experiments. Each model was trained and tested on both the conventional human figure subset and the Four-Armed Avalokiteshvara dataset, ensuring that the evaluation covered both standard and complex multi-limb cases. All comparative experiments were conducted under identical settings, including keypoint definitions, training protocols, and evaluation metrics, to ensure fairness and consistency in performance comparison across different models and categories.

In these comparative experiments, we used the same dataset and ensured fairness by maintaining consistent experimental conditions, including batch size, learning rate, and image resolution. Additionally, multiple common evaluation metrics for pose estimation tasks were selected, namely mAP@0.5, mAP@0.75, mAP@0.5:0.95, PCK@0.1, and OKS. Among them, mAP@0.5, mAP@0.75, and mAP@0.5:0.95 represent the average precision at IoU thresholds of 0.5, 0.75, and from 0.5 to 0.95 with a step size of 0.05, providing a comprehensive assessment of model accuracy. PCK@0.1 evaluates keypoint localization accuracy by measuring whether keypoints fall within 10% of the target bounding box size. OKS measures the distance between predicted keypoints and ground-truth keypoints while weighting keypoint visibility, making it a more robust metric for evaluating Thangka images, where clothing occlusions and complex poses are common.

As shown in Table 1, MythPose achieves consistent and notable improvements over the best-performing baseline models across all evaluation metrics for human pose estimation in Thangka images. For mAP@0.5, MythPose achieves a score of 89.13%, exceeding the strongest baseline by 5.75%. This improvement demonstrates MythPose’s superior capability in accurate keypoint detection and localization, which is mainly due to the Mamba-based ASSB module that effectively captures global spatial relationships and contextual dependencies crucial in Thangka compositions. For mAP@0.75, MythPose attains 73.54%, surpassing the best baseline by 2.3%. This stricter metric reflects precise localization performance, and the observed gain highlights the contribution of the dual attention feature fusion module, which enables adaptive integration of detailed local and high-level semantic features. Regarding mAP@0.5:0.95, MythPose reaches 66.07%, outperforming the top baseline by 2.29%. This advantage across a range of IoU thresholds indicates the robustness of our approach to varying levels of pose complexity and occlusion, resulting from the synergy between global and multi-scale feature modeling. In terms of PCK@0.1, MythPose achieves 92.51%, with a 3.19% improvement over the best baseline. This metric, which measures keypoint localization accuracy within a small spatial tolerance, benefits significantly from the Keypoint Association Loss that enforces precise grouping and reduces keypoint confusion, especially in multi-limb and occluded cases. Finally, on the OKS metric, MythPose attains 87.22%, exceeding the strongest baseline by 2.18%. This demonstrates the model’s enhanced robustness to occlusion and variability in appearance, which is further supported by our explicit keypoint grouping mechanism and advanced feature fusion strategies. Collectively, these results confirm that the architectural innovations and targeted loss functions in MythPose directly address the core challenges of Thangka pose estimation, enabling the best performance across all aspects of keypoint detection. Furthermore, as presented in Table 2, we conduct additional evaluations on the Four-Armed Avalokiteshvara dataset, which presents greater challenges due to its complex multi-limb configurations. On this more difficult subset, the performance of all models, including MythPose, shows a general decline compared to conventional human pose estimation, reflecting the increased difficulty of multi-limb keypoint detection in Thangka art. Despite this, MythPose consistently achieves the highest scores across all evaluation metrics, demonstrating clear improvements over the best-performing baselines. These results confirm that our model not only achieves leading performance on standard human figures but also maintains a strong robustness and generalization ability under complex multi-limb conditions in artistic images.

To further test the generalization ability of our model, we conducted experiments on the Human-Art dataset [59], which also contains a wide variety of artistic images. As shown in Table 3, MythPose achieves the best results on all evaluation metrics. Our model reaches the highest mAP@0.5 of 94.48%, which is better than all baseline methods. For mAP@0.75 and mAP@0.5:0.95, MythPose also obtains the top scores of 78.23% and 63.71%. In addition, MythPose achieves 95.83% for PCK@0.1 and 92.51% for OKS, again surpassing all compared models. These results show that our method works very well not only on Thangka images, but also on other challenging artistic datasets. This demonstrates the strong generalization and robustness of MythPose for human pose estimation in diverse artistic styles.

**Figure 6:**
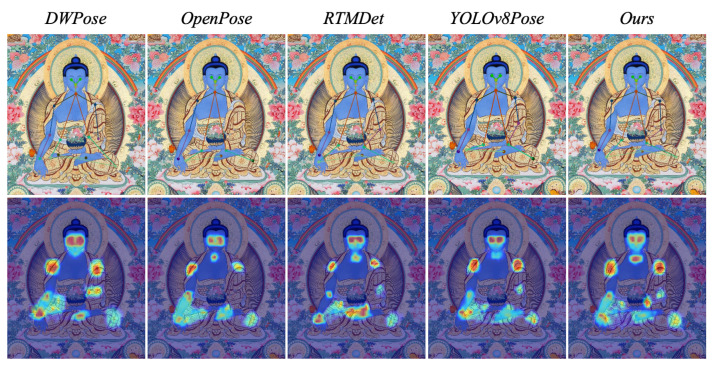
Each column represents the results from a different model: DWPose, OpenPose, RTMDet, YOLOv11Pose, and our proposed method. The **first row** shows the human pose estimation results for each model applied to Thangka images, while the **second row** displays the corresponding heatmaps, where the color intensity indicates keypoint confidence—areas closer to red signify higher attention and confidence in keypoint localization. Our method demonstrates improved keypoint localization with more focused and clearer heatmap responses compared to the other models.

Figure 6 visualizes the model’s attention regions during human pose estimation using Grad-CAM, where red areas indicate higher attention weights. All models primarily focus on the head and hands. However, for Thangka images, MythPose adjusts its attention toward symmetrical limb features, such as the shoulders and hands. These heatmaps demonstrate that MythPose effectively learns the unique structural characteristics of Thangka figures and focuses more accurately on keypoint regions compared to other models.

MythPose outperforms all comparative models across all evaluation metrics, demonstrating superior accuracy and stability in human pose estimation for Thangka images. This improvement is attributed to the introduction of the Mamba structure in YOLOv11-Pose, which enhances global feature extraction. Additionally, the feature fusion module integrates shallow and deep features, while the KAL loss function mitigates the issue of keypoint confusion in multi-limb figures.

### 4.5. Ablation Study

**Figure 7:**
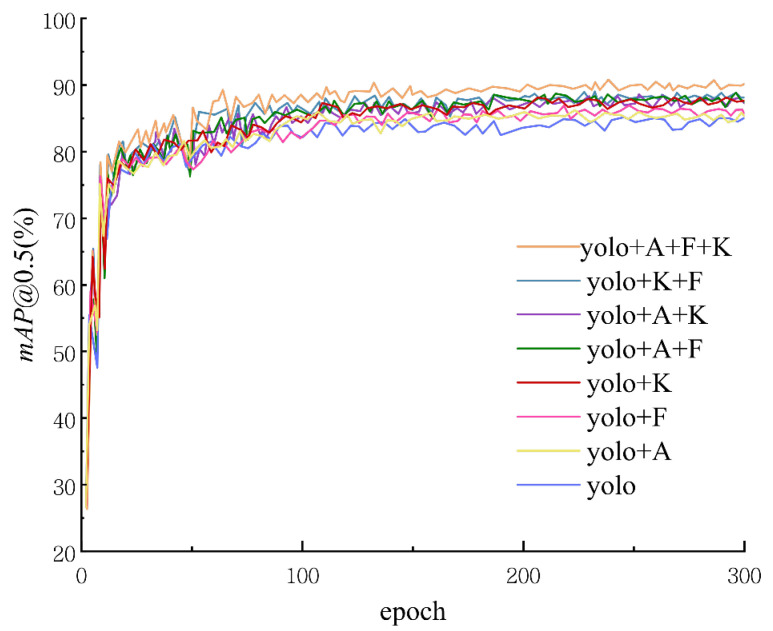
The graph shows the training results of our ablation experiments, with mAP@0.5 (%) as the performance metric over 300 epochs. Different configurations of our model are compared, including combinations of the ASSB module (A), the fusion module (F), and KAL loss (K) alongside the baseline YOLO model. The results highlight the impact of each component on model performance, with certain combinations achieving higher and more stable mAP values compared to the baseline.

To investigate the performance contribution of each module proposed in this paper for the Thangka human pose estimation task, ablation experiments were designed to test the effectiveness of ASSB, the feature fusion module, and KAL, as well as their combined contributions. The training results of the ablation experiments are shown in Figure 7, where the results indicate that mAP@0.5:0.95 stabilizes after approximately 300 training epochs.

The evaluation metrics selected for the ablation experiments include precision, recall, and mAP@0.5:0.95. These metrics effectively reflect the contribution of each module to the model’s performance. The results of the ablation experiments are summarized in Table 4, where the (✓) symbol indicates whether a component is included.

Table 4 illustrates the impact of each component in our model, including ASSB, feature fusion, and KAL, on performance metrics such as Precision, Recall, and mAP@0.5. The baseline model, without any added components, achieves a Precision of 83.42%, Recall of 81.23%, and mAP@0.5 of 83.38%. Adding ASSB alone improves the Precision to 84.53%, Recall to 82.14%, and mAP@0.5 to 86.21%, demonstrating a substantial performance boost. Incorporating feature fusion individually raises Precision further to 84.87%, Recall to 82.47%, and mAP@0.5 to 85.52%. When KAL is introduced as the sole enhancement, the model achieves a Precision of 85.04%, Recall of 82.73%, and mAP@0.5 of 85.83%

Combining ASSB and feature fusion results in a Precision of 85.32%, a Recall of 83.06%, and an mAP@0.5 of 87.13%, while pairing ASSB with KAL yields an even better Precision at 85.65%, Recall at 83.34%, and mAP@0.5 at 87.42%. The combination of feature fusion and KAL produces a Precision of 85.36%, a Recall of 82.51%, and an mAP@0.5 of 86.72%. Our best results are achieved when all three components—ASSB, feature fusion, and KAL—are combined. This configuration attains a Precision of 86.07%, a Recall of 84.22%, and an impressive mAP@0.5 of 89.13%, representing notable improvements of 2.65%, 3.99%, and 5.75% in Precision, Recall, and mAP@0.5, respectively, compared to the baseline.

As shown in the results of Table 4, the ASSB, feature fusion module, and KAL components proposed in this paper contribute to the performance improvement of the model. Specifically, ASSB enhances the model’s performance by introducing the Mamba structure, the feature fusion module effectively integrates deep and shallow features, and KAL improves the accuracy of keypoint detection. The synergy of these three components significantly boosts the model’s detection performance. The results in the table clearly demonstrate both the individual and synergistic performance of each module and show that the model achieves the highest values in all metrics when all three components are included, thus validating the effectiveness of the complete model.

## 5. Discussion

The human pose estimation task in Thangka images faces several unique challenges, such as image damage, unpredictable numbers of limbs, overlapping regions, and occluded clothing. These issues impose high demands on keypoint detection accuracy and model robustness. To address these difficulties, we constructed a comprehensive Thangka keypoint dataset containing both normal and multi-limbed figures, and we applied various data augmentation methods to enrich the data and improve generalization.

Building upon YOLOv11n, our proposed MythPose model incorporates the Mamba structure—replacing the Bottleneck units in the C3k2 modules—to enhance the extraction of global features and capture long-range dependencies. A feature fusion module is further introduced before the detection head to combine shallow and deep features, thereby boosting detection accuracy. Additionally, a keypoint association loss function is designed to improve the model’s ability to distinguish between overlapping or similar limbs.

Experimental results show that MythPose achieves significant improvements in keypoint detection accuracy compared to the baseline methods. Our model enables a more reliable and fine-grained localization of keypoints, even in the presence of partial occlusion, color similarity, or complex limb configurations. This provides valuable support for the analysis of artistic proportions and stylistic evolution in Thangka paintings and contributes to the broader goal of digital preservation and research in Tibetan art.

Despite these advancements, some limitations remain. The model’s performance can degrade in cases of severe color fading or substantial image damage, as commonly observed in ancient Thangka paintings. In such scenarios, pose estimation becomes less reliable due to the loss of visual cues. Future work will focus on improving the robustness of MythPose to damaged or low-quality images—potentially by incorporating restoration modules, additional preprocessing techniques, or leveraging multi-modal information.

In summary, MythPose offers a practical and effective solution for human pose estimation in Thangka images and provides new insights for complex keypoint detection tasks in other forms of traditional or artistic imagery. However, the current work is limited by the lack of publicly available annotated datasets for other relevant art forms, which restricts direct validation of the model’s cross-domain generalizability. In future work, we plan to expand data collection efforts to include additional types of religious and mural art, and further explore the transferability and adaptation of MythPose to broader cultural heritage applications. We believe that our approach can inspire new research directions in pose estimation and structural analysis across diverse artistic and cultural contexts.

## Figures and Tables

**Table 1:** Performance comparison of different models on the conventional human figures keypoint detection task.

Model	mAP@0.5	mAP@0.75	mAP@0.5:0.95	PCK@0.1	OKS
DWPose [53]	78.62%	65.34%	57.42%	85.23%	81.14%
OpenPose [54]	80.13%	67.85%	59.31%	86.71%	82.39%
RTMDet [55]	81.57%	68.92%	61.15%	88.43%	83.72%
AlphaPose [56]	82.02%	69.10%	61.00%	88.72%	84.02%
TokenPose [28]	81.89%	69.85%	61.58%	89.01%	83.87%
ViTPose++ [57]	82.31%	69.97%	62.22%	88.94%	84.26%
YOLOv11-Pose [58]	83.38%	71.24%	63.78%	89.32%	85.04%
**MythPose**	**89.13%**	**73.54%**	**66.07%**	**92.51%**	**87.22%**

**Table 2:** Performance comparison of different models on the Four-Armed Avalokiteshvara (multi-limb) keypoint detection task.

Model	mAP@0.5	mAP@0.75	mAP@0.5:0.95	PCK@0.1	OKS
DWPose [53]	76.12%	62.48%	54.05%	82.93%	78.24%
OpenPose [54]	77.51%	64.02%	55.88%	84.41%	79.85%
RTMDet [55]	78.63%	65.34%	57.11%	85.97%	81.13%
AlphaPose [56]	78.22%	64.91%	56.35%	85.41%	80.52%
TokenPose [28]	78.75%	65.02%	56.60%	85.47%	81.08%
ViTPose++ [57]	78.81%	65.17%	56.73%	85.67%	81.34%
YOLOv11-Pose [58]	79.82%	66.10%	57.94%	86.34%	81.92%
**MythPose**	**86.10%**	**69.68%**	**61.45%**	**89.32%**	**84.02%**

**Table 3:** Performance comparison of different models on the Human-Art dataset.

Model	mAP@0.5	mAP@0.75	mAP@0.5:0.95	PCK@0.1	OKS
DWPose [53]	87.03%	67.29%	53.12%	91.19%	87.11%
OpenPose [54]	86.55%	65.48%	51.46%	89.24%	85.57%
RTMDet [55]	90.48%	71.22%	55.27%	92.12%	89.51%
AlphaPose [56]	87.54%	66.46%	52.08%	90.13%	86.77%
TokenPose [28]	92.34%	74.57%	59.39%	92.49%	90.53%
ViTPose++ [57]	92.67%	75.53%	60.44%	93.32%	91.02%
YOLOv11-Pose [58]	92.25%	75.49%	59.07%	92.54%	89.57%
**MythPose**	**94.48%**	**78.23%**	**63.71%**	**95.83%**	**92.51%**

**Table 4:** Ablation experiments for each component in our model on the conventional human figure keypoint detection dataset.

ASSB	Feature Fusion	KAL	Precision (%)	Recall (%)	mAP@0.5 (%)
-	-	-	83.42	81.23	83.38
✓	-	-	84.53	82.14	86.21
-	✓	-	84.87	82.47	85.52
-	-	✓	85.04	82.73	85.83
✓	✓	-	85.32	83.06	87.13
✓	-	✓	85.65	83.34	87.42
-	✓	✓	85.36	82.51	86.72
✓	✓	✓	**86.07**	**84.22**	**89.13**

## Data Availability

The datasets generated and/or analyzed during the current study are not publicly available due to concerns related to cultural sensitivity and image authorization agreements. However, the data are available from the corresponding author upon reasonable request.

## Abbreviations

The following abbreviations are used in this manuscript:

ASSB	Advanced Scanning and Synthesis Block
ASS	Advanced Scanning and Synthesis
DPA	Dual-Pool Attention
DW Conv	Depthwise Convolution
GAP	Global Average Pooling
GAN	Generative Adversarial Network
GMP	Global Max Pooling
HRNet	High-Resolution Network
KAL	Keypoint Association Loss
SAA	Spatial Axis Attention
SSM	Selective State Space Model
UASM	Unified Attention Synergy Module
